# Hospital Expenditure.—II

**Published:** 1902-08-23

**Authors:** 


### The Institutional Workshop.

### HOSPITAL EXPENDITURE.—II.

By the Author of "The Hospital Commissariat."

We have shown in a previous article that in attempting to draw comparisons between the expenditure of various institutions it is necessary to have an accurate knowledge of the persons who are boarded within its walls.

To know how many men and women are catered for in addition to the patients is the beginning of just criticism, but it is only the beginning.

A hundred circumstances may modify the expenditure of a large institution household, just as they modify the expenditure of each separate household in a London street, and it would not be safe to conclude that A. is more extravagant than B. because he spends more on a particular article, unless A. has been challenged to explain the difference and has failed to give a satisfactory reason.

One of the most obvious causes of different expenditure in Metropolitan hospitals is to be found in the cost of what in-patients provide for themselves. At some hospitals, tea, sugar, and butter are provided by the patients' friends for them, and the saving on these articles, even reckoning that a good proportion of the patients on low diet do not receive them, may be reckoned at £2 per bed. In some hospitals the patients are charged for laundry. It is probable that they are nowhere charged the full cost, but the saving effected will stand for about 30s. to £2 per bed. And a corresponding deduction in the miscellaneous domestic expenses will be shown in institutions where brushes, combs, towels, soap, etc., are brought by the patients.

The saving is not always apparent in the institution where these simple and rather old-fashioned demands are made on the patients. For example, C. may provide everything for the patients, and yet show a lower cost per bed on provisions than D. But then D. may board a larger proportion of nurses than C., and this item in the housekeeping being heavier in proportion than the cost of tea and butter, will outweigh considerably the saving which D. effects by the articles which the patients provide.

Another very obvious cause of difference in hospital housekeeping is shown in the very different class of patients received at these institutions. Hospitals which receive for the most part acute cases, and are provided with convalescent homes into which they may draft their patients as soon as they can be moved, will always have a large proportion of their patients on low or simple diet. The cost of low diet, which consists mainly of milk, varied by beef-tea, is not more than about 4d. a day. But in the case of a hospital in a manufacturing district, either in London or elsewhere, which receives a number of accident cases, there will be a large proportion of semi-convalescent men on full diet, with rations of beef and beer, costing 8d. or 10d. a day.

The difference in cost is at once apparent. In the same way a difference of sex or class in the "persons other than patients" boarded in the establishment may make an astonishing difference in the bills. Supposing E. to have its own laundry, its engine house, and bakery, it will then keep engineers, etc., always on the premises in addition to porters and messengers. If these receive board, an allowance of malt liquor will in all probability be included, and the bill for beer will seem very high in comparison with F., where board wages (with allowance for beer reckoned under the head of salaries) are given to the men about the place, or G., where no laundry is maintained.

Again, a special class of beds will affect expenditure very materially. At Guy's Hospital the cost of food in the Bright Ward, where paying patients are received, is about three times that in the public wards of the hospital. In the Middlesex Hospital the same is true of the patients in the cancer wards, who are provided practically with anything they can eat. And at the Westminster Hospital the same is no doubt true of the incurable wards. Speaking generally, the longer the average stay of the patients in the hospital, the higher the cost of provisions, for it is in the earlier stages of treatment for the most part that low diet is in force.

All sorts of unexpected conditions are brought to light when a little patience is devoted to the causes which govern hospital expenditure. Not very long ago attention was directed to the unusually large butcher's bill of the London Hospital in proportion to its household, and it was then ascertained that the wards there set aside for Jewish patients entailed heavy extra expense on "kosher" meat, which had to be specially killed and cooked in the manner prescribed by the patient's religion.

Lastly, it must be borne in mind that the closing of wards for cleaning or other purposes for any long period exercises a marked influence on the cost of each patient and the cost of each occupied bed. The number of patients accommodated during the year and the average number of occupied beds is decreased by the reduction of ward space; all the routine expenses go on as before, and divided up among the smaller number the effect is to show an increase of expenditure under every heading. It is true that if half the hospital is closed for three months a good number of the nurses will be granted a long holiday, and a saving will be effected on their board and that of the patients. But the same organisation required for 400 patients will be needed for 200, and it will be vain to expect any substantial reduction of expense to balance the lowered average. Hence in drawing comparisons between the expenditure per bed of the same hospital in different years it is necessary to ascertain whether in each year the establishment was in full working order, and make full allowance for any interruption to the work.

We have touched very briefly on some of the varying conditions in voluntary hospitals. It is by no means an exhaustive survey, and we believe that no single hospital is without some special circumstance making for cost, either below or above the average, which should be understood before judgment is passed.

We believe that in all cases hospitals benefit by showing confidence in the public and giving a very careful account of the manner in which the funds entrusted to them are spent. But we are quite sure that much harm is done when hasty and ill-balanced conclusions are published, drawn from bare outlines of hospital expenditure. There is no graver fault than wantonly to cast a slur on good men's work. We would urge all who are dissatisfied to go to the fountain-head for information, and only after the most careful investigation to proceed to the last extremity and make public the adverse judgment which they have formed, in full possession of all surrounding circumstances.

